# Validity of Measurement of Shear Modulus by Ultrasound Shear Wave Elastography in Human Pennate Muscle

**DOI:** 10.1371/journal.pone.0124311

**Published:** 2015-04-08

**Authors:** Naokazu Miyamoto, Kosuke Hirata, Hiroaki Kanehisa, Yasuhide Yoshitake

**Affiliations:** National Institute of Fitness and Sports in Kanoya, Kagoshima, Japan; Rensselaer Polytechnic Institute, UNITED STATES

## Abstract

Ultrasound shear wave elastography is becoming a valuable tool for measuring mechanical properties of individual muscles. Since ultrasound shear wave elastography measures shear modulus along the principal axis of the probe (i.e., along the transverse axis of the imaging plane), the measured shear modulus most accurately represents the mechanical property of the muscle along the fascicle direction when the probe’s principal axis is parallel to the fascicle direction in the plane of the ultrasound image. However, it is unclear how the measured shear modulus is affected by the probe angle relative to the fascicle direction in the same plane. The purpose of the present study was therefore to examine whether the angle between the principal axis of the probe and the fascicle direction in the same plane affects the measured shear modulus. Shear modulus in seven specially-designed tissue-mimicking phantoms, and in eleven human in-vivo biceps brachii and medial gastrocnemius were determined by using ultrasound shear wave elastography. The probe was positioned parallel or 20° obliquely to the fascicle across the B-mode images. The reproducibility of shear modulus measurements was high for both parallel and oblique conditions. Although there was a significant effect of the probe angle relative to the fascicle on the shear modulus in human experiment, the magnitude was negligibly small. These findings indicate that the ultrasound shear wave elastography is a valid tool for evaluating the mechanical property of pennate muscles along the fascicle direction.

## Introduction

Determination of individual muscles’ material/mechanical properties along the long axis (i.e., main axis) of the muscle belly allows a better understanding of muscle function. Recently, ultrasound elastography has been used to quantify mechanical properties along the long axis of in-vivo human muscles. Unlike qualitative ultrasound elastography (e.g., strain imaging [1,2]), ultrasound shear wave elastography has an advantage in that it is simple to use and objectively quantifies tissue mechanical properties along the long axis based on the propagation speed of remotely induced shear waves [3,4,5,6].

**Fig 1:**
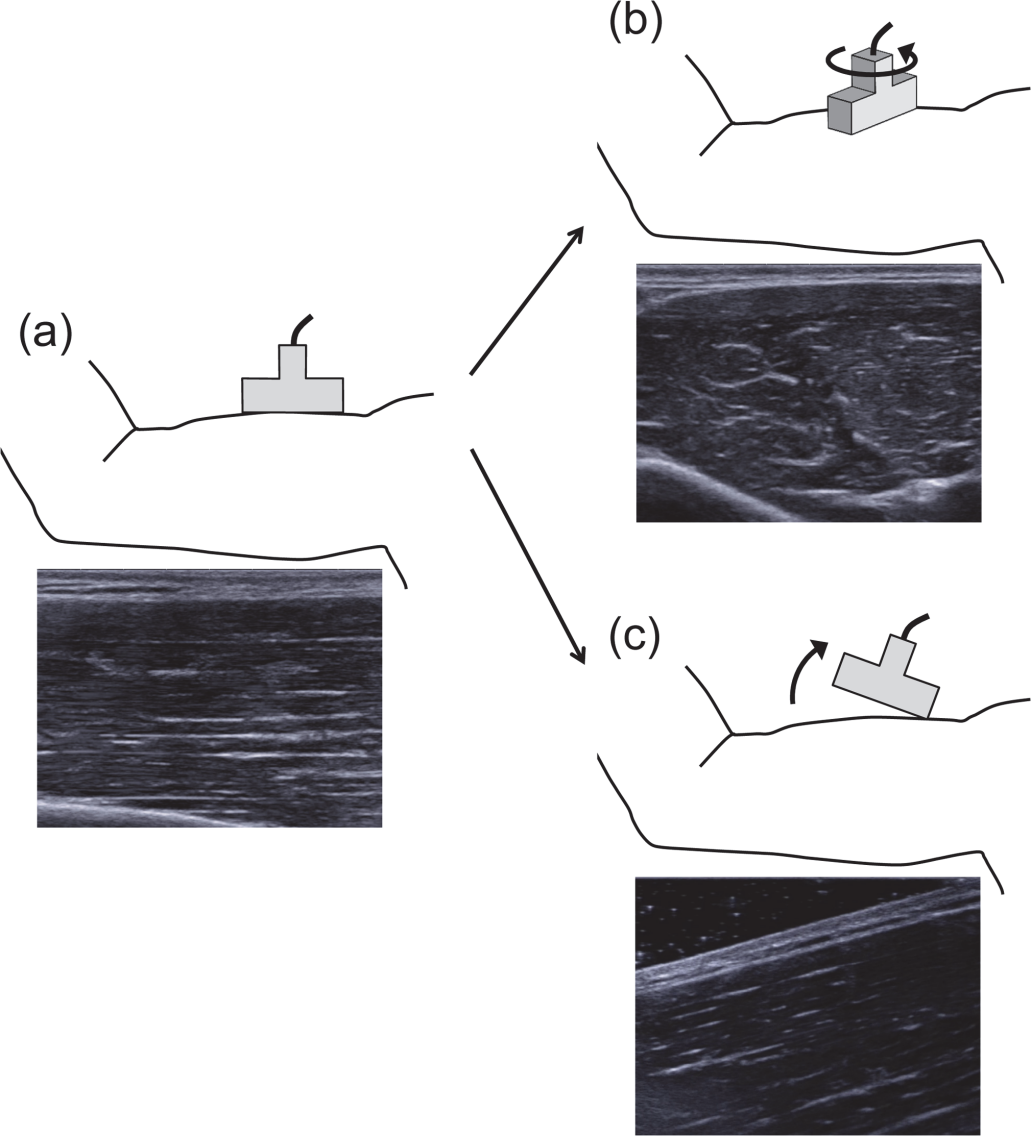
Examples of probe position and corresponding ultrasound B-mode image. (a) The probe is placed parallel to the fascicle direction in the same plane of the ultrasound image of the fusiform biceps brachii. (b) The probe is rotated relative to the fascicle plane. (c) The probe is rotated relative to the fascicle plane as end-to-end fascicles are observed in the measurement plane.

An ex-vivo study with the gastrocnemius and tibialis anterior of fresh roaster chickens showed a linear relationship between the shear modulus measured by ultrasound shear wave elastography and passive muscle force [7]. Similarly, Eby et al. have reported on the brachialis whole-muscle of swine that shear modulus measured by ultrasound shear wave elastography was highly correlated with measured values of Young’s modulus obtained via traditional materials testing [8]. Additionally, the shear modulus has been reported to be in high correlation with the active muscle force observed during voluntary isometric contractions of the first dorsal interosseous and abductor digiti minimi [9]. The aforementioned findings suggest that ultrasound shear wave elastography can be a valuable tool for measuring mechanical properties of individual muscles. However, some studies have provided evidence indicating that particular attention should be paid to interpreting shear wave elastography data obtained from pennate muscles [10,11]. Ultrasound shear wave elastography measures shear wave speed and corresponding shear modulus along the principal axis of the probe (i.e., along the transverse axis of the imaging plane), not along the muscle fiber/fascicle direction. Thus, the measured shear modulus most accurately represents the mechanical property of the muscle along the fascicle direction when the probe is placed parallel to the fascicle direction in the same plane of the ultrasound image (Fig 1A). In other words, when targeting pennate muscles such as the gastrocnemius [11,12] and vastus lateralis [12,13], the shear modulus measured by ultrasound shear wave elastography cannot accurately represent the mechanical properties of muscle along the fascicle direction.

With regards to the issue mentioned above, previous studies have indicated that shear modulus is sensitive to the probe position with respect to direction of the fascicle plane (Fig 1B) [10,11]. For example, Gennisson et al. have shown that the shear modulus measured by shear wave elastography decreased as the rotation angle of the probe with respect to the fascicle plane was increased in the fusiform human biceps brachii [10]. Although the previous studies have shown the anisotropic effect of a skeletal muscle by rotating the probe obliquely with respect to the plane of the fascicle when measuring the shear modulus of muscle, the end-to-end fascicles were not in the measurement plane (Fig 1B). What should be considered when measuring shear modulus along the fascicle direction in pennate muscles in vivo is the effect of the probe angle relative to the fascicle direction in the same plane (Fig 1C). Therefore, the purpose of the present study was to elucidate whether the probe angle relative to the fascicle direction in the same plane affects the shear modulus measured by ultrasound shear wave elastography.

## Materials and Methods

### Study design

The present study consisted of two experiments. The main purpose of the first experiment (phantom experiment) was to examine the effect of boundary condition (i.e., ultrasound gel angle between the probe and object surface). In the second experiment (human experiment), we investigated the effect of the probe angle relative to the fascicle for the fusiform biceps brachii (BB) and the pennate medial gastrocnemius (MG) muscles. In both experiments, a real-time ultrasound shear wave elastography system (Aixplorer Ver. 4.3.2, Supersonic Imagine, France) with a 4–15 MHz linear probe (50 mm width; SL15-4, Supersonic Imagine, France) was used. Shear modulus was calculated from the shear wave propagation speed along the transverse axis of the imaging plane. In the present study, a single examiner performed all measurements.

### Phantom experiment

The effect of the probe angle relative to the surface of phantoms was assessed. This was performed by measuring the shear modulus of seven tissue-mimicking ultrasound phantoms with shear modulus ranging from 6 kPa to 73 kPa (10.0 cm × 6.0 cm × 2.5 cm; OST, Japan). The manufacturer made the isotropic phantoms from acrylic hydrogel with high-water content and calibrated the values by a conventional stress-strain test. The shear modulus of these phantoms were similar to the acoustic properties of human soft tissues [14]. In order to examine the effect of boundary condition, the probe was adjusted so that the surface of phantoms across the B-mode image was parallel to the horizontal or 20°-oblique lines drawn on the monitor, with sufficient amount of ultrasound gel between them. The selection of this angle was for the final purpose of clarifying the effect of the probe angle to the fascicle on the shear modulus in in-vivo human muscles, of which pennation angles such as the pennate triceps surae [15] and quadriceps femoris [16] were near the angle in the anatomical position. Care was taken not to press and deform the phantom while scanning. For each condition, the shear modulus was measured three times, and the mean value was used for further analysis. The coefficient of variation (CV) in the three measurements was < 1.2%. To ensure test-retest repeatability, trials were performed two times for each condition. Furthermore, to examine day-to-day repeatability, the same procedures were repeated on two different days with an interval of 1 day in-between.

### Human experiment

Eleven healthy male subjects (1.71 ± 0.04 m, 70.7 ± 12.7 kg, 22.2 ± 1.1 years; mean ± SD) participated in this study. The subjects were fully informed of the procedures as well as the purpose of the study. They were instructed to refrain from strenuous physical activity 24 h before testing. Written informed consent was obtained from each subject. Subject information was anonymized prior to analyses. This study was approved by the Ethics Committee on Human Research of National Institute of Fitness and Sports in Kanoya and performed in accordance with the Declaration of Helsinki.

**Fig 2:**
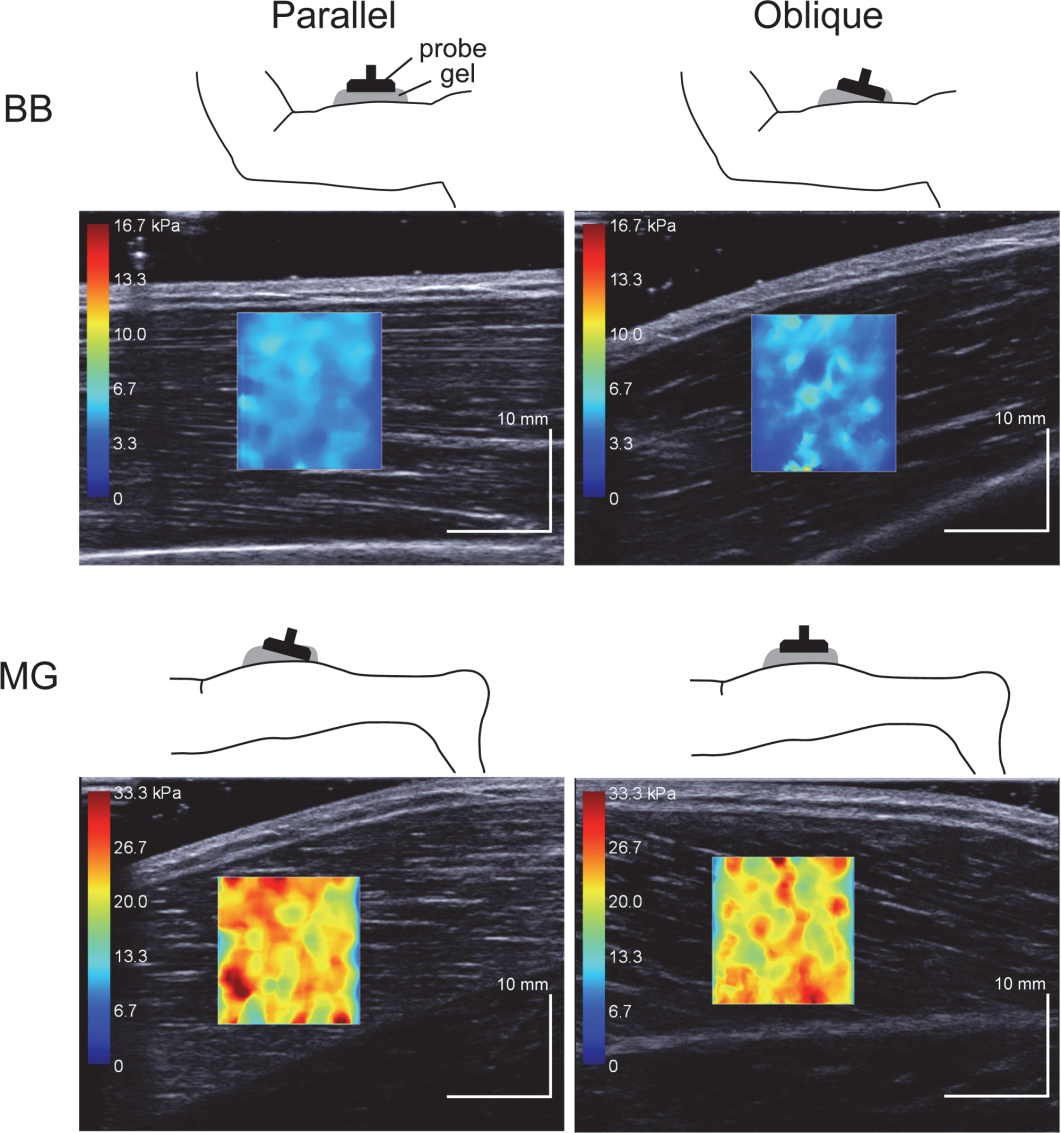
Examples of shear elastic modulus measurements of the biceps brachii (BB) and medial gastrocnemius (MG). Left: Parallel condition, Right: Oblique condition. The colored region represents the shear elastic modulus map with the scale to the left of the figure.

We targeted BB and MG as the representatives of fusiform and pennate muscles, respectively. In BB measurement, subjects lay supine on a dynamometer bed (CON-TREX MJ, PHYSIOMED, Germany) with their shoulders abducted at 90°. Their forearm was kept in a neutral position. The measurement was performed at three different elbow joint angles of 90°, 115°, and 140° (180° = full extension). In MG measurement, subjects lay prone on the bed with their right knees fully extended. The right foot was firmly strapped to the dynamometer footplate. The measurements were performed at three different ankle angles of the anatomical position (defined as 0°, with larger numbers for dorsiflexion), 10°, and 20°. The subjects were instructed to fully relax their legs and arms throughout the measurements. In each measurement, the ultrasound probe was placed over the muscle belly of BB and MG. The probe orientation was adjusted to identify several fascicles without interruption across the B-mode image in a certain plane. Then, the probe was positioned parallel or obliquely to the skin surface, with sufficient amount of ultrasound gel between them. Namely, when the probe was positioned parallel to the skin surface, BB fascicles were visualized horizontally, whereas MG fascicles ran obliquely across the B-mode image (Fig 2). In the other condition, the probe was tilted to visualize several fascicles parallel to the 20°-oblique line across the B-mode image for BB measurement, whereas the probe was adjusted for the fascicles to run horizontally for MG measurement (Fig 2). Care was taken not to press and deform the muscles while scanning. In the present study, we refer to measurements in which the probe and fascicles were parallel as the Parallel condition (see Fig 2 left) and that in which the probe was positioned obliquely to the fascicles as the Oblique condition (see Fig 2 right). For each condition, the measurements of the shear modulus were conducted three times (CVs < 1.9% and 2.8% for Parallel and Oblique conditions, respectively), and the mean value was used for further analysis.

### Data and statistical analysis

The spatial average of shear modulus in the selected circular area (13–15 mm diameter) was computed by using software in the ultrasound system. The reason for selecting the nonconstant diameter was to ensure the largest area but exclude aponeurosis and subcutaneous adipose tissue from the analysis for each subject. In order to evaluate the test-retest (intra-day) and inter-day repeatability of phantom measurements, the CVs, intra-class correlation coefficients (ICCs), and absolute differences were calculated. A paired t-test was performed to compare the shear modulus between Parallel and Oblique conditions. For data obtained from human experiments, separate two-way ANOVAs (Joint angle (3) × Probe angle (2)) with repeated measures were used for BB and MG data. The CVs, ICCs, and absolute differences were calculated. The significant level for all comparisons was set at *P* < 0.05. All the statistical analyses were performed with statistical software (SPSS Statistics 21, IBM Japan, Tokyo, Japan). Data are expressed as means and SDs.

## Results

### Phantom experiment

Table 1 shows the CVs, ICCs, slopes of the regression line, and absolute differences between trials (intra-day) and between 2 days (inter-day) for the shear modulus in both Parallel and Oblique conditions. The intra- and inter-day CVs for the Parallel condition were 1.1% and 1.0%, respectively, and 1.1% and 0.8%, respectively, for the Oblique condition. The ICC(1,2) were high with the regression line close to the identity line for each condition (*P* < 0.001). The intra- and inter-day absolute differences were 0.3 kPa and 0.4 kPa for the Parallel condition, respectively, and 0.2 kPa and 0.3 kPa, respectively, for the Oblique condition. A paired t-test detected no significant difference between Parallel and Oblique conditions (*P* = 0.487). The absolute difference was 0.5 ± 1.0 kPa.

**Table 1:** Repeatability of the shear elastic modulus in Phantom study.

	CV (%)	ICC	Slope	Absolute difference (kPa)
Test-retest				
Parallel	1.1 ± 0.9	1.00	1.01	0.3 ± 0.3
Oblique	1.1 ± 0.6	1.00	1.02	0.4 ± 0.5
Day-to-day				
Parallel	1.0 ± 0.4	1.00	0.99	0.2 ± 0.2
Oblique	0.8 ± 0.6	1.00	1.00	0.3 ± 0.4

CV: coefficients of variation, ICC: intra-class correlation coefficients, Slope: slopes of the regression line.

### Human experiment

**Fig 3:**
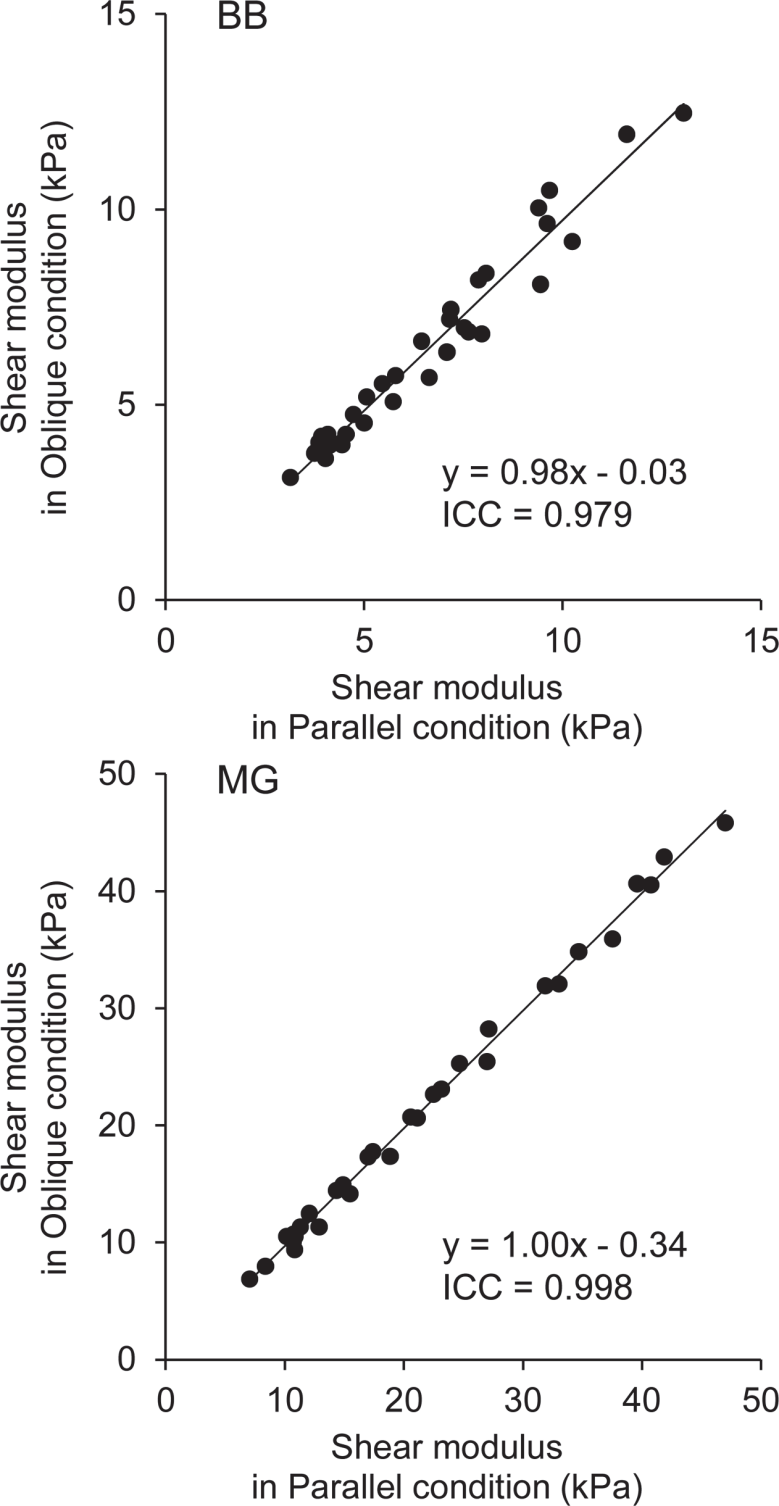
Scatter plots of shear elastic modulus between Parallel and Oblique conditions.

For BB data, in the Parallel condition, the intra-day CV, ICC, and absolute difference were 1.6%, 1.00, and 0.4 kPa, respectively (Table 2). In the Oblique condition, these values were 2.7%, 1.00, and 0.5 kPa, respectively. Furthermore, the regression lines obtained from the ICC analysis were close to the identity line in both conditions. A two-way ANOVA revealed significant main effects of Joint angle (*P* < 0.001, observed power = 1.000) and Probe angle (*P* = 0.048, observed power = 0.528) with no significant interaction (*P* = 0.230). The ICC(1,2) between Parallel and Oblique conditions was 0.979 (*P* < 0.001; Fig 3). The absolute difference between Parallel and Oblique conditions was 0.5 ± 0.6 kPa.

**Table 2:** Repeatability of the shear elastic modulus in Human study.

	CV (%)	ICC	Slope	Absolute difference (kPa)
BB				
Parallel	1.6 ± 1.2	1.00 ± 0.00	1.04 ± 0.05	0.4 ± 0.4
Oblique	2.7 ± 1.0	1.00 ± 0.00	0.99 ± 0.05	0.5 ± 0.7
MG				
Parallel	2.1 ± 1.9	1.00 ± 0.00	1.02 ± 0.02	0.4 ± 0.4
Oblique	2.7 ± 1.0	1.00 ± 0.00	1.01 ± 0.04	0.5 ± 0.7

BB: biceps brachii, CV: coefficients of variation, ICC: intra-class correlation coefficients, MG: medial gastrocnemius, Slope: slopes of the regression line.

For MG data, in the Parallel condition, the intra-day CV, ICC, and absolute difference were 2.1%, 1.00, and 0.4kPa, respectively. In the Oblique condition, these values were 2.7%, 1.00, and 0.5 kPa, respectively. Furthermore, the regression lines obtained from the ICC(1,2) analysis were close to the identity line in both conditions (Table 2). A two-way ANOVA revealed significant main effects of Joint angle (*P* < 0.001, observed power = 1.000) and Probe angle (*P* = 0.020, observed power = 0.704) with no significant interaction (*P* = 0.697). The ICC between Parallel and Oblique conditions was 0.998 (*P* < 0.001; Fig 3). The absolute difference between Parallel and Oblique conditions was 0.6 ± 0.5 kPa.

## Discussion

To the best of our knowledge, the present study is the first case to examine the effect of the probe tilting angle relative to the fascicle direction in the same imaging plane (Fig 1C) on the shear modulus measured by the ultrasound shear wave elastography. The major findings of the present study are that 1) no influence of the probe angle was observed in isotropic phantoms, and 2) although there was an effect of the probe angle relative to the fascicle on the shear modulus in human muscles, the magnitude was negligibly small (< 1.3% of the measured values). The present findings indicate that the ultrasound shear wave elastography is a valid tool for evaluating the mechanical properties of pennate muscles along the fascicle direction, assuming the probe angle is no more than 20°.

Before interpreting the present results, it should be noted as to why the ultrasound shear wave elastography is appropriate at all in the first place for conducting shear modulus measurements in muscle. As previously described in detail [3,5], the ultrasound shear wave elastography system in the present study uses a transient and remote mechanical vibration generated by an acoustic radiation force to perturb muscle tissues. The beam generates a remote vibration that results in the propagation of a transient shear wave. An ultrafast echographic imaging is performed to capture the shear wave propagation at a very high frame rate (up to 20 kHz), and then to estimate the shear wave propagation speed (c) along the principal axis of the probe with one-dimensional cross-correlation algorithm. Assuming a linear elastic behavior, the shear modulus (μ) was calculated using c as follows:

$$\text{μ}=\text{ ρ}�c^{2}$$

where ρ is the density of skeletal muscle. As for the experimental validity of measurement of shear modulus by using the ultrasound shear wave elastography, Yoshitake et al. have shown with phantom experiments that when the shear modulus measured by the ultrasound shear wave elastography was compared with the values calibrated by traditional stress-strain testing, the ICC was very high (0.999), with the regression line very close to the line of identity (slope = 1.012, y-intercept = -5.447 kPa) [14]. Additionally, Eby et al. have already examined in an ex-vivo study and reported that shear modulus measured by the ultrasound shear wave elastography was highly correlated with Young’s modulus measured by traditional materials testing [8]. These findings indicate that shear wave elastography should be a valid tool for assessing the elasticity of soft tissue such as human muscles.

As for the experimental repeatability of measurements of shear modulus, Yoshitake et al. have reported a very high value of the intra-day ICC (0.98) for BB [14]. Lacourpaille et al. have observed an intra-day ICC of 0.95 with the CV of 4.6% for the resting MG [12]. Similarly, Taniguchi et al. have also shown with the resting human medial and lateral gastrocnemius that the intra-day ICC was very high (0.97–0.98), with CV and standard error of measurement values less than 4% and 0.2 kPa, respectively [17]. These are similar to the values in human experiment of the present study. However, the corresponding data obtained in the phantom experiment of the present study was much better. This is probably because skeletal muscle is an anisotropic and inhomogeneous structural tissue from the viewpoint of its microstructure. Taking into account the previous and present findings, we can conclude that the measurement of shear modulus for either in-vivo human fusiform or pennate muscle can be performed with high repeatability using ultrasound shear wave elastography.

Previous studies, which examined the effect of aligning the probe obliquely with respect to the plane of the fascicle in the fusiform human biceps brachii (Fig 1B), showed that the shear modulus measured by ultrasound shear wave elastography significantly decreased as the rotation angle increased [10,11]. For example, according to the finding of Maïsetti et al., the shear modulus measured with the probe rotation angle of 20° relative to the fascicle plane has been shown to be lower by approximately 30–40% than that measured with the probe placed parallel to the fascicle [11]. On the other hand, the present study examined whether the probe angle relative to the fascicle direction in the same plane affects the shear modulus measured and showed a smaller effect (< 1.3%) of the probe angle (i.e., pennation angle). The difference between the present and previous findings is possibly due to the fact that a skeletal muscle is an anisotropic tissue. It has been shown that the shear waves preferably propagate along the fiber/fascicle direction [18,19]. However, the muscle anisotropy does not substantially play a role in the present study since the probe was tilted in the fascicle plane. Another possibility for the discrepancy between the theory and our finding regarding the effect of probe angle relative to the fascicle is the difference in boundary condition (i.e., ultrasound gel angle) between the probe and skin surface. However, this was rejected based on the present finding of the phantom experiment that there was no substantial difference in the shear modulus between Parallel and Oblique conditions. Taken together, although the precise reasons for the discrepancy in shear modulus in pennate muscles between the theoretical (i.e., the cosine effect of the probe angle due to one-dimensional cross-correlation algorithm (cos20°) is about 6%) and present values (< 1.3%) remain unclear, it is reasonable to conclude that the shear modulus between the Parallel and Oblique conditions in the present study are relatively equal.

A limitation associated with the present study is that the shear modulus was not measured for contracting muscles. As mentioned above, the repeatability of measuring shear modulus for resting muscles was very high. For contracting muscles, the intra-day ICC was slightly low with greater CV in BB (ICC = 0.95, CV = 7–13%) [20]. Since we were concerned that sample variability could mask potential differences in shear modulus between conditions, the shear modulus was measured only for resting muscles. Therefore, further investigations are warranted to reveal whether similar findings could hold true for contracting muscles (i.e., greater shear modulus). Another limitation is that the probe angle relative to the fascicle was restricted to 20°. The pennation angle of MG has been reported to be greater when contracting as well as in the knee or plantar flexed position [15]. The inherent measurable zone of the elastography apparatus used in the present study was up to approximately 30 mm beneath the probe surface. In our preliminary phantom experiment, when the probe angle relative to phantom surface was set at more than 20° with great amount of ultrasound gel, the area for measuring shear modulus was very small. Moreover, in the case of in-vivo human muscles, since there is approximately 10 mm layer of skin and subcutaneous tissues between the probe and muscle, the measurement area was practically nonexistent.

In conclusion, the present study revealed that although there was a significant effect of the probe angle relative to the fascicle on the shear modulus in human muscles, the difference was negligibly small. The findings obtained here suggest that the ultrasound shear wave elastography is a valid tool to evaluate the mechanical properties of pennate muscles along the fascicle direction if the probe angle (which is close to pennation angle) is no more than 20°. In terms of clinical practice, the current results support future use of the ultrasound shear wave elastography, such as for assessment of tonus, contracture, and the effects of different treatments (e.g., passive stretching, electrical stimulation, and tendon lengthening surgery), in not only the fusiform but also the pennate muscles.
